# The Prevalence of Barrett’s Esophagus in Outpatients with Dyspepsia in Shaheed Beheshti Hospital of Kashan

**Published:** 2013-09

**Authors:** Tahere Khamechian, Javad Alizargar, Tahere Mazoochi

**Affiliations:** Anatomical Sciences Research Center, Kashan University of Medical Sciences, Kashan, Iran

**Keywords:** Barrett’s esophagus, Endoscopy, Heartburn, Pathology

## Abstract

Gastroesophageal reflux disease (GERD) is the main known etiological factor for Barrett’s esophagus (BE), and BE is the precursor lesion of esophageal adenocarcinoma. The prevalence of BE is reported mostly from gastroenterology centers and there are only a few reported cases from outpatients with dyspepsia. A large number of patients with GERD have degrees of dyspepsia. This study primarily aimed to determine the prevalence of BE in dyspeptic patients. Outpatients with dyspepsia who referred to our Endoscopy Unit for endoscopy were included in this study. Esophageal biopsy was performed by an endoscopist, and BE diagnosis was established based on the abnormal appearance of the distal esophagus in endoscopy and also based on the presence of intestinal metaplasia in pathologic examination. The prevalence of BE was 5.4% (based on endoscopy) and 3.7% (based on pathology). Sixty-nine percent of the patients with confirmed BE were younger than 50 and 31% were over 50 years of age. Eighty-one percent of the patients with confirmed BE reported GERD symptoms as their dominant dyspepsia symptom, whereas only 20.4% of those without BE reported GERD symptoms (P<0.001). Additionally, BE had a relatively high prevalence in our dyspeptic patients. The high prevalence of GERD symptoms in BE underscores the need for endoscopy for dyspeptic patients.

## Introduction

The prevalence of gastroesophageal reflux disease (GERD) continues to rise along with the prevalence of Barrett’s esophagus (BE) and esophageal adenocarcinoma.^1^ GERD is the main known etiological factor for BE, which is the precursor lesion of esophageal adenocarcinoma.^2^ Defined as the change in the lining of the distal esophagus, BE can be recognized with endoscopy and be documented by the presence of *goblet *cells and other criteria for intestinal metaplasia (IM) in biopsies taken during endoscopy.^3^ Hiatus hernia, obesity, and presence of helicobacter pylori in the gastrointestinal tract are some of the risk factors for BE.^4^ These factors are believed to amplify BE by increasing acid reflux. Many gastroenterologists make the diagnosis of BE via endoscopy and confirm it with the presence of IM in biopsies obtained from the esophagus.^2^ The criterion for endoscopy is the presence of chronic GERD after the consumption of proton-pump inhibitors or acid suppressors for at least 4 weeks.^5^ The association between BE and adenocarcinoma is the principal factor that drives physicians to evaluate GERD patients endoscopically.^6^^-^^9^ In terms of prevalent, BE is found in 2% of the adult population and 3-5% of GERD patients.^2^ The overall prevalence of BE in patients with chronic GERD is between 3 and 12%.^6^^,^^8^^,^^9^

The prevalence of BE has been reported mostly from gastroenterology centers, and there have been a few reported cases from outpatients with dyspepsia. The likelihood of the coexistence between GERD and dyspepsia in a large number of patients highlights the need to evaluate dyspeptic patients for BE.^8^ Endoscopy is widely used for diagnosing BE; be that as it may, the exact risk factors of BE and efficacy of endoscopy in diagnosing BE have yet to be fully elucidated. This present report was aimed specifically at determining the prevalence of BE in dyspeptic outpatients and exploring the potential risk factors for its presence. It also sought to determine the efficacy of gastrointestinal (GI) endoscopy for BE diagnosis in a selected population.

## Patients and Methods

This is a prospective study on the outpatients of our Gastrointestinal Clinic. The study population comprised patients who were over 18 years old and had a primary complaint of dyspepsia of at least 3 months’ duration (intermittent or continuous). The study was approved by the Ethics Committee of Kashan University of Medical Sciences and was conducted between 2007 and 2011.

Dyspepsia was defined as a complex of discomfort or pain in the epigastric region (with or without acid regurgitation), excessive burping or belching, abdominal bloating, early satiety, or feeling of abnormal or slow digestion or heartburn.^8^ A documented history of upper GI surgery, clinical investigation of dyspepsia by endoscopy or radiology (in the previous 6 months) or on more than two occasions in the past 10 years, and use of proton-pump inhibitors within 30 days or H2-receptor antagonists within 14 days of enrolment were the exclusion criteria of the present study.

Out of all the outpatients originally enrolled, those who provided oral consent to an endoscopic examination were recruited in our study and were referred to the Endoscopy Unit of Shaheed Beheshti Hospital, a central hospital in the Iranian city of Kashan. Data on the recruited outpatients’ age, sex, nationality, weight and height, presence and dominance of GERD symptoms, and duration of dyspeptic symptoms were recorded in separate forms. The presence of BE was assessed in two ways: endoscopically and histologically. Diagnosis of BE was established based on the abnormal appearance of the distal esophagus in endoscopy. If there was a suspicion of Barrett’s epithelium in the distal portion of the esophagus, the endoscopist determined the case as BE and the case was marked as a “BE case by endoscopy”. The presence of gastric-appearing mucosa or columnar-lined esophagus was the criterion for the endoscopist’s report of BE. The lengths of the abnormal epithelium were not recorded. Biopsies from all the cases were taken just proximal to the gastroesophageal junction, according to the standard practice for histological confirmation during the procedure. The decision as regard the number of biopsies to be obtained was made upon the approximation of the Barrett’s epithelium length by the endoscopist. If the pathologist observed evidence of IM in the biopsies, BE could be confirmed and the case was marked as a “BE case by pathology”. These data were added to the patient’s form. The data were entered into SPSS software and analyzed using descriptive statistics as well as the chi-square test and *t* test.

## Results

Of the 1,156 outpatients originally enrolled, 12 patients did not consent to have endoscopy. These 12 patients were comprised of 9 Afghans, who failed to return for endoscopy for unknown reasons, and 3 Iranian patients, who decided that endoscopy was unnecessary despite having received thorough explanation about the necessity of the modality. A total of 1,144 dyspeptic patients, consisting of 1,100 (96.2%) Iranian and 44 (3.8%) Afghan patients at a mean age of 45.2 years old, underwent endoscopy. BE was diagnosed endoscopically in 62 (5.4%) and pathologically in 42 (3.7%) cases. All these 42 cases were “BE cases by endoscopy” as well, while 20 (32.2%) cases that were “BE cases by endoscopy” were not confirmed as “BE cases by pathology”. Thus, the sensitivity of endoscopy for the diagnosis of BE was 100% but its specificity was 67.8%. The mean age of the patients with confirmed BE was 53.2 years. In terms of gender, 42.6% of the patients without BE were male and 57.4% were female, whereas 64.3% of the patients with BE were male and 35.7 were female (P=0.005) (table1). 

**Table 1:** Demographic characteristics of patients with and without BE

**BE**		**N**	**Y**	**P value**
Sex	Male	470 (42.6)	27 (64.3)	0.005
	Female	632 (57.4)	15 (35.7)	
Nationality	Iranian	1060 (96.2)	40 (95.2)	0.753
	Afghan	42 (3.8)	2 (4.8)	
Age	Mean	44.95	53.21	<0.001
	SD	14.89	12.60	
BMI	Mean	28.86	28.85	0.995
	SD	4.76	4.85	

N: no; Y: yes; SD: Standard deviation; BMI: Body Mass Index

Hiatus hernia was diagnosed in 10.2% of all the patients (117 out of 1,144). In addition, 9.1% of the patients without IM had hiatus hernia, while 40.5% of the patients with IM had hiatus hernia (P<00.1) (table 2). Reflux esophagitis was detected in 54.8% of the patients with BE and in only 4.4% of the patients without BE (P=0.003). 

The mean duration of dyspeptic symptoms in the 42 BE patients was 10.29 years; six (14.3%) patients reported symptoms of less than 5 years’ duration and 1 (2.4%) patient reported symptoms of over 1 year’s duration. A comparison between those with and without BE revealed that the patients with BE had longer periods of dyspepsia (P<0.01) (table 2).

**Table 2:** Comparisons of risk factors of BE between patients with and without BE

**BE**		**N**	**Y**	**P value**
HH	N	1002 (90.9)	25 (59.5)	<0.001
	Y	100 (9.1)	17 (40.5)	
Dyspepsia years	Mean	4.42	10.29	<0.001
	S.D	4.24	6.43	
Heartburn	N	824 (74.8)	6 (14.3)	<0.001
	Y	278 (25.2)	36 (85.7)	
Dominant heartburn	N	877 (79.6)	8 (19)	<0.001
	Y	225 (20.4)	34 (81)	
Reflux esophagitis	N	115 (10.4)	19 (45.2)	0.003
	Y	49 (4.4)	23 (54.8)	

HH: Hiatus hernia; N: no; Y: yes; SD: standard deviation

Of the 1,144 patients, 314 (27.4%) cases had acid regurgitation or heartburn and 259 had these symptoms as their dominant symptom. Out of these 259, 34 (13.1%) cases had BE. Thirty-four out of the 42 (81%) patients with confirmed BE reported either heartburn or acid regurgitation as their most bothersome (dominant) dyspepsia symptom, compared with 225 (20.4%) of the 1,102 patients without BE (P<0.001) (table 2).

The mean Body Mass Index (BMI) among all the 1,140 patients was 28.8, and there were no significant differences between the patients with confirmed BE and those without BE (P=0.995).

## Discussion

The accepted method for diagnosing BE is observing IM in biopsies taken from the esophagus. There is a debate as to whether the presence of gastric metaplasia (without IM) should classify a patient as having BE or not. In this study, the presence of IM in pathology was the key point to classify a patient as having BE. 

In our 1,144 uninvestigated dyspepsia outpatients- who underwent endoscopy, the prevalence of BE was 5.4% on the basis of the endoscopic suspicion of gastric metaplasia in the distal esophagus and 3.7% on the basis of the histological confirmation of the diagnosis by the presence of IM. In one single-center study on 1,248 Iranian GERD patients, the prevalence of endoscopically suspected and pathologically confirmed BE was 8.3% and 2.4%, respectively.^10^ It should be noted that dyspeptic patients, rather than GERD patients, were investigated in the present study. The prevalence of BE among the patients with acid regurgitation and heartburn (GERD symptoms) as their dominant symptom was 13.1% in our study; this is relatively higher than the figures reported by previous Iranian investigations^10^^,^^11^ and is also higher than the reported 3-12% in other studies.^6^^,^^8^^,^^9^ Nevertheless, the prevalence rate in our study was lower than the 24.1% reported in a study conducted in Japan.^12^ On study reported that hiatus hernia and esophagitis were more common in patients with BE and BE was more prevalent in males and older ages.^13^ In our study, those complaining of heartburn or acid regurgitation tended to have BE more frequently than their counterparts in other similar studies. BE based on endoscopy was reported at 0.3% to 2% in a study on 3,634 Canadian patients, and only a minority (0.3%) was histologically confirmed.^14^ This may reflect the prevalence of BE in our area. Indeed, in our study, 67% of the endoscopically diagnosed cases of BE were confirmed by histology. This rate was 11% in another study.^15^ These results highlight the significant role of the experience of an endoscopist in diagnosing BE. 

It has been previously shown that both longer durations and severity of heartburn are risk factors for the development of the adenocarcinoma of the distal esophagus. Patients with BE in the current study reported dyspepsia symptoms of longer durations and only 14.3% had symptoms of less than 5 years’ duration. BE is likely to cause GERD; it is, therefore, advisable that people undergo endoscopy at least once in their lifetime.^8^

## Conclusion

In summary, the overall prevalence of histologically confirmed BE was 3.7% in our outpatients with dyspepsia. Moreover, in the patients with dominant symptoms of heartburn, the prevalence of BE was 13.5%. These data could be drawn upon in the discussion on the need for a once-in-a-lifetime endoscopy in patients with dyspeptic symptoms. Our results suggest that if endoscopy is recommended and indeed performed at an older age (such as age>50 years) and in patients with symptoms of more than 5 years’ duration, it would augment the yield of the diagnosis of BE.
